# Type I-F CRISPR-Cas resistance against virulent phages results in abortive infection and provides population-level immunity

**DOI:** 10.1038/s41467-019-13445-2

**Published:** 2019-12-04

**Authors:** Bridget N. J. Watson, Reuben B. Vercoe, George P. C. Salmond, Edze R. Westra, Raymond H. J Staals, Peter C. Fineran

**Affiliations:** 1 Department of Microbiology and Immunology, University of Otago, Dunedin, 9054 New Zealand; 2 Department of Biochemistry, University of Cambridge, Cambridge, CB2 1QW UK; 3 ESI, Biosciences, University of Exeter, Cornwall Campus, Penryn, TR10 9FE UK; 4 Laboratory of Microbiology, Wageningen University and Research, 6708 WE Wageningen, The Netherlands; 5 Bio-Protection Research Centre, University of Otago, Dunedin, New Zealand; 6 Present Address: ESI, Biosciences, University of Exeter, Cornwall Campus, Penryn, TR10 9FE UK

**Keywords:** Bacteriophages, CRISPR-Cas systems, Phage biology

## Abstract

Type I CRISPR-Cas systems are abundant and widespread adaptive immune systems in bacteria and can greatly enhance bacterial survival in the face of phage infection. Upon phage infection, some CRISPR-Cas immune responses result in bacterial dormancy or slowed growth, which suggests the outcomes for infected cells may vary between systems. Here we demonstrate that type I CRISPR immunity of *Pectobacterium atrosepticum* leads to suppression of two unrelated virulent phages, ɸTE and ɸM1. Immunity results in an abortive infection response, where infected cells do not survive, but viral propagation is severely decreased, resulting in population protection due to the reduced phage epidemic. Our findings challenge the view of CRISPR-Cas as a system that protects the individual cell and supports growing evidence of abortive infection by some types of CRISPR-Cas systems.

## Abstract (web summary)

Bacteria use CRISPR-Cas systems to protect themselves against viral infections. Here, Watson et al. show that a type I CRISPR-Cas system can induce abortive viral infection, where infected cells do not survive but viral propagation is decreased, thus protecting the bacterial population.

## Introduction

To respond to the pressure of phage infection, bacteria have evolved various lines of defence^1–3^. The adaptive arm of these defences is provided by CRISPR-Cas, which provides immunity through CRISPR RNA guided cleavage of phage genomes^4,5^. CRISPR-Cas systems are incredibly diverse and are currently classified into two major classes (1 and 2), six types (I–VI) and >30 subtypes^6,7^ (for recent reviews, see refs. ^4,5,8^). Crucially, recent studies revealed that at least some CRISPR-Cas variants, belonging to types VI and III, induce cell dormancy through collateral RNA cleavage following target recognition^9–13^. Furthermore, it is possible that type V systems induce cell death through ssDNA cleavage^14^. In contrast, the most abundant type I CRISPR-Cas systems, which make up around 60% of all CRISPR-Cas systems^15^, as well as the somewhat less common type II systems, can increase the survival of infected individuals^16,17^. Yet type I immunity against some phages resulted in population decline^18^. Hence, it appears that immunity mediated by type I systems may lead to different outcomes for infected cells.

Here we examine the outcome of CRISPR-Cas immunity using *Pectobacterium atrosepticum*, which carries a type I–F system, and two unrelated virulent phages as model systems. We find that CRISPR-Cas immunity reduces the number of cells that release phages and of those that produce progeny, the burst size is decreased. Infected cells do not survive phage infection, yet they reduce phage amplification, which provides protection at the population level. In abortive infection (Abi) systems, phages can adsorb, but phage replication is interrupted, leading to the death of the infected cell and the release of few, or no phages^19,20^. Consequently, the bacterial population survives^19,20^. Therefore, the observed CRISPR-Cas immunity phenotype to virulent phage infection entirely fulfils the definition of abortive infection. This has key implications for the way natural selection operates on CRISPR-Cas^21^ and is analogous to that observed for other kin-selected altruistic defences, which also provide population-level benefits despite the death of the infected individuals.

## Results

### CRISPR-Cas reduces phage infectious centres and burst size

**Fig. 1:**
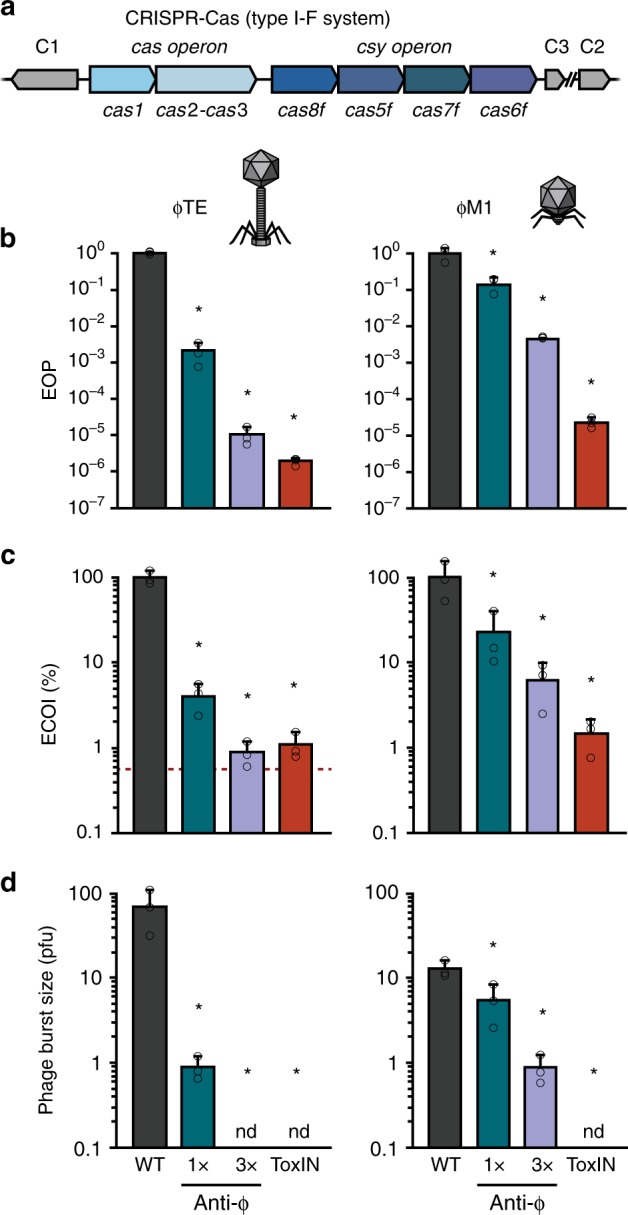
CRISPR-Cas reduces phage infectious centres and burst size. **a** Schematic of the type I–F system in *P. atrosepticum*. C1, C2 and C3 represent CRISPR arrays 1, 2 and 3. **b** Phage resistance (i.e. efficiency of plating (EOP)), **c** efficiency of centre of infection (ECOI) formation and **d** average burst size was assessed for the phage-sensitive WT, anti-ɸ strains with one (1×) or three (3×) spacers targeting ɸTE and ɸM1 and cells with ToxIN. In **c** the red dashed line represents the background level of phages (in the other panels the limit of detection is below the axis limits). Phages were added at a multiplicity of infection (MOI) of 0.1 for both **c** and **d**. Data shown is the mean + one standard deviation (SD). nd, not detected. Statistical significance was calculated using one-way ANOVA using Dunnett’s multiple comparison test, comparing strains with targeting spacers to the control with no-targeting spacers. No significance was detected, unless indicated (*p ≤ 0.05). All data are provided in Supplementary Table 1. Source data are provided as a Source Data file.

To investigate the outcomes of phage infection in the presence of CRISPR-Cas immunity, we examined the response to phage infection by *P. atrosepticum*, which contains a type I–F system (Fig. 1a)^22,23^. We used two different phages, ɸTE and ɸM1, members of the *Myoviridae* and *Podoviridae* families, respectively. Phage infectivity was assessed using strains with one or three phage-targeting spacers in the chromosomal CRISPR arrays and compared with *P. atrosepticum* lacking phage-targeting spacers. CRISPR-Cas provided protection against ɸTE and ɸM1 infection, reducing the efficiency of plating (EOP) by at least 10-fold with one spacer, with additional spacers increasing resistance to 10^5^-fold with three spacers (Fig. 1b, Supplementary Table 1). To determine what stage of phage reproduction was impeded, we investigated the effects of CRISPR-Cas on defined aspects of infection. CRISPR-Cas caused a decrease in the efficiency of centre of infection (ECOI) formation (Fig. 1c), meaning that for ɸTE, only 4 or 1% of infected cells released at least one infectious phage (for the 1 × and 3 × anti-ɸTE strains, respectively). Following ɸM1 infection, only 23 or 6% of cells released phages (for 1 × and 3 × anti-ɸM1, respectively). Next, one-step growth curves were performed to observe phage growth on the resistant hosts (Supplementary Fig. 1 and Supplementary Table 1). The average phage burst size was determined for each host and the number was significantly reduced by CRISPR-Cas (Fig. 1d). For ɸTE, both the 1 × or 3 × anti-ɸTE strains almost completely suppressed the burst and for ɸM1 it was reduced by >90% on the 3× anti-ɸM1 strain. As expected, adsorption was unaffected by CRISPR immunity (Supplementary Fig. 1 and Supplementary Table 1). Therefore, the *P. atrosepticum* type I–F CRISPR-Cas immunity reduced both the number of cells releasing phages and the average number of phages released per cell.

We previously characterised an Abi system in *P. atrosepticum*, ToxIN, which functions as a toxin-antitoxin system^24,25^. ToxIN provides protection against both ɸTE and ɸM1 phages, acting as an Abi system, so we included ToxIN to compare the phenotypes provided by CRISPR-Cas and Abi immunity genes^24–26^. The ToxIN Abi system provided strong phage protection, reducing the EOP by 10^6^ and 10^5^-fold against ɸTE and ɸM1, respectively (Fig. 1b). For both phages, only 1% of phage-infected cells harbouring ToxIN released any new viral progeny (Fig. 1c) and the average burst size was undetectable (Fig. 1d). As expected for a post-adsorption phage resistance mechanism, ToxIN had no effect on adsorption (Supplementary Table 1). The outcomes of ToxIN and CRISPR-Cas-mediated immunity on the different aspects of infection were therefore qualitatively similar with respect to phage adsorption and amplification.

### The type I–F CRISPR-Cas system does not enable cell survival

**Fig. 2:**
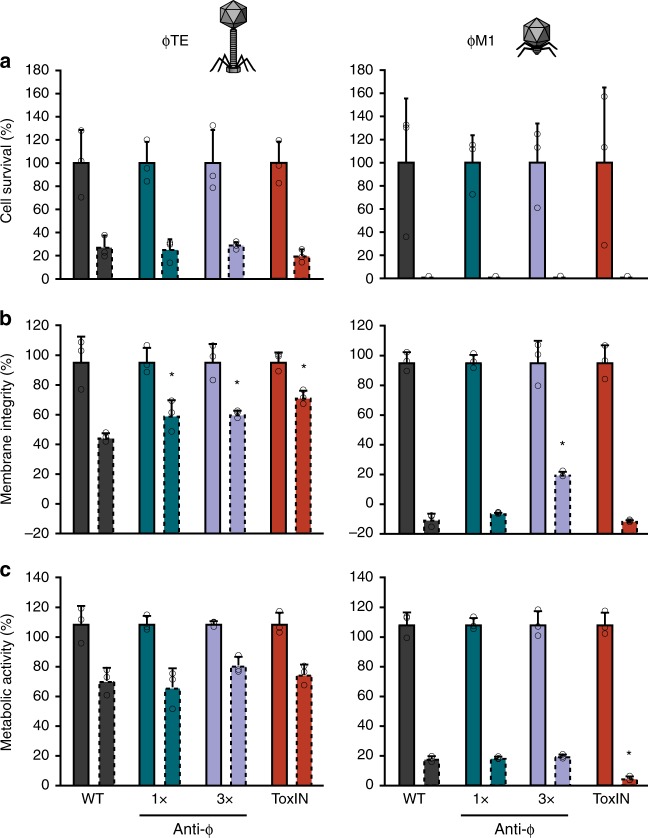
The type I–F CRISPR-Cas system does not enable survival of infected cells. **a** Cell survival was assessed for the WT, 1× and 3× anti-ɸ strains, and ToxIN, using both ɸTE and ɸM1 (infected at an MOI of 2). **b** The percentage of cells with intact membranes was determined using LIVE/DEAD^™^ staining and **c** the percentage of metabolically active cells was assessed using the resazurin dye. For **b** and **c** cells were infected at an MOI of 2.5. Solid outline bars represent mock infected samples, dashed outline bars represent phage-infected samples. Data shown are the mean + one SD. Statistical significance was calculated using one-way ANOVA using Dunnett’s multiple comparison test, comparing strains with targeting spacers to the control with no-targeting spacers. No significance was detected, unless indicated (*p ≤ 0.05). Source data are provided as a Source Data file.

Next, we assessed cell survival of bacteria with CRISPR-Cas immunity upon infection with the virulent phages. Surprisingly, CRISPR-Cas immunity provided no enhancement in cell survival measured in single-cell viable count assays compared with the phage-sensitive WT or the ToxIN Abi system (Fig. 2a), regardless of the multiplicities of infection (MOI) that were used (Supplementary Fig. 2A). To further investigate cell survival, we assessed membrane integrity and cellular metabolic activity of phage-infected cells (Fig. 2b, c, Supplementary Fig. 2B and C). Phage infection led to significant reductions in both membrane integrity and cellular metabolism even in the presence of CRISPR-Cas or ToxIN immunity. As a control, surface mutants (i.e. bacteria carrying mutations in the phage receptor genes on the bacterial cell surface) were isolated that were resistant to either phage. As expected for adsorption inhibition, surface resistance against either phage resulted in cells retaining membrane integrity and metabolic activity upon phage challenge, but not when challenged with a phage that uses a different receptor (Supplementary Fig. 3). Together, we see that phage adsorption is not affected, but rather phage replication is interrupted by CRISPR-Cas, which results in fewer phage progeny and leads to the death of the infected cell. Hence the immunity seen with the type I–F system in *P. atrosepticum* fulfils the definition of abortive infection^19,20^.

### Increased CRISPR-Cas resistance does not enhance cell survival

**Fig. 3:**
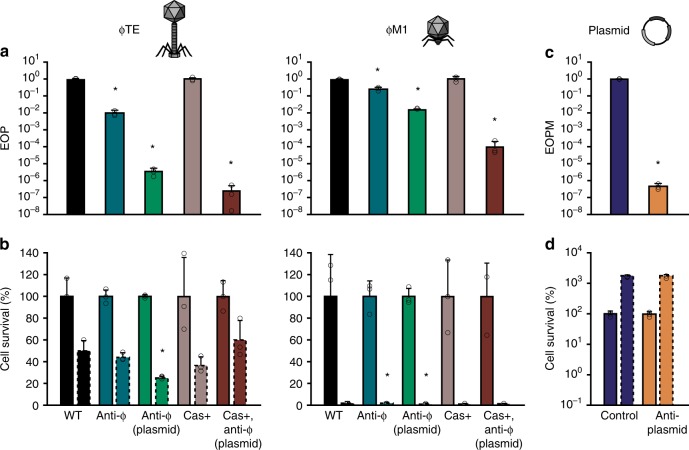
CRISPR-Cas overexpression increases phage resistance but infected cells do not survive. **a** Phage resistance (EOP) and **b** cell survival was assessed for WT (with empty vector, pPF975) “WT”, 1× anti-ɸ (PCF190 for ɸTE/PCF254 for ɸM1 (with empty vector), chromosomally expressed) “anti-ɸ”, 1× anti-ɸ plasmid expressed (WT carrying pPF1423 for ɸTE/pPF1421 for ɸM1) “anti-ɸ (plasmid)”, Cas overexpression (PCF610 (with empty vector)) “Cas+” and Cas overexpression with 1× anti-ɸ plasmid expressed (PCF610, pPF1423 for ɸTE/pPF1421 for ɸM1) “Cas+, anti-ɸ (plasmid)”. Solid outline bars represent mock infected samples, dashed outline bars represent phage-infected samples. **c** Efficiency of plasmid maintenance (EOPM) and **d** cell survival was assessed for strains carrying pTargeted (with the *expI* gene) and pControl (inducible mini-CRISPR array with no anti-*expI* spacer) “Control” or pTargeted and pCRISPR (anti-*expI* spacer) “anti-plasmid”. Solid outline bars represent CRISPR repressed samples, dashed outline bars represent CRISPR induced samples. Data shown are the mean + one SD. Statistical significance was calculated using one-way ANOVA using Dunnett’s multiple comparison test, comparing strains with targeting spacers to the control with no-targeting spacers. The Cas overexpression and Cas overexpression with 1× anti-ɸ plasmid expression strains, as well as the strains in **d**, were compared using an unpaired T-test. No significance was detected, unless indicated *p ≤ 0.05). Source data are provided as a Source Data file.

One possible explanation why CRISPR-Cas did not promote survival following infection, resulting in abortive infection, could be due to an insufficient immune response, leading to incomplete phage clearance. For example, anti-CRISPR proteins might reduce defence and prevent effective phage clearance^27,28^. However, the fact that abortive infection occurred with two unrelated phages and we could not bioinformatically detect known anti-CRISPRs argues against this theory. Another possibility is that the CRISPR-Cas components are limiting. Indeed, since the phage-targeting spacers are in CRISPR arrays that carry 30 (CRISPR1) and 11 (CRISPR2) other spacers, most effector complexes will be loaded with non-phage-targeting crRNAs. To explore if an increased abundance of Cas complexes loaded with phage-targeting crRNAs would result in survival of infected cells, phage-targeting spacers were overexpressed from plasmids in the presence or absence of Cas overexpression (Fig. 3). Increased phage-targeting crRNAs significantly boosted phage resistance compared with chromosomal expression, and induction of Cas expression further enhanced resistance, by up to ~10^4^–10^7^ fold compared to the WT (Fig. 3a). However, no marked restoration in cell survival was detected compared with the sensitive WT strain (Fig. 3b).

While these data show that CRISPR-immune bacteria do not survive virulent phage infection even under artificially high CRISPR expression levels, it is unclear whether this is due to cell death induced by CRISPR-Cas components (analogous to the dormancy observed for type VI systems^10^), or due to the phage, which may express lethal genes prior to clearance of the infection. To explore this question, we examined the outcome of targeting plasmid DNA for the cells with CRISPR-Cas immunity (Fig. 3c, d). The *P. atrosepticum* CRISPR-Cas system effectively inhibits transformation and conjugation^29^, but those assays fail to assess the outcome for cells eliciting effective CRISPR immunity since they are killed by the antibiotic. To directly test whether plasmid targeting by the I–F system reduces cell survival in *P. atrosepticum*, we induced a mini-CRISPR array with a spacer targeting a plasmid and assessed total cell counts and plasmid loss. Plasmid targeting decreased cells bearing the plasmid by 10^6^-fold in 18 h but did not decrease total cell numbers. Hence, these experiments show that the combination of both phage infection and CRISPR-Cas targeting is required for abortive infection, since cells survived plasmid targeting.

### CRISPR-Cas provides population protection at low phage doses

**Fig. 4:**
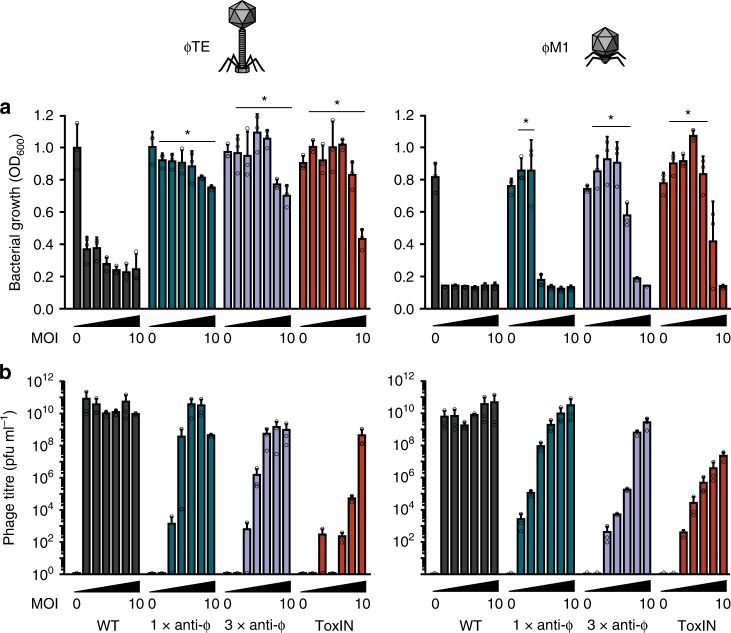
Populations of anti-ɸ strains only grow at low phage doses. WT, 1× and 3× anti-ɸ strains, and ToxIN were grown with phages added at a range of doses (MOIs: 0 (buffer), 10^−4^, 10^−3^, 10^−2^, 10^−1^, 1 and 10). **a** The final bacterial growth levels (OD_600_) and **b** the final phage titres were determined after 16 h. Data shown are the mean + one SD. Statistical significance was calculated using one-way ANOVA using Dunnett’s multiple comparison test, comparing strains with targeting spacers to the control with no-targeting spacers. No significance was detected, unless indicated (*p ≤ 0.05). Source data are provided as a Source Data file.

Even though abortive infection results in the death of the infected individual, these defence systems may be favoured by natural selection because of their population-level benefits if these are predominantly directed at clone mates (i.e. kin selection). To explore these kin-selected benefits, we compared population growth of cells carrying CRISPR-Cas or ToxIN (Abi) under increasing phage pressures (increasing MOIs) (Supplementary Fig. 4). Phage-sensitive WT *P. atrosepticum* populations were susceptible to phages at any MOI. The phage effects on population growth were stronger and faster with increasing phage numbers, but even with an MOI of 0.0001, WT populations collapsed (Fig. 4a). As predicted for an Abi mechanism, cultures containing ToxIN grew with low phage doses, but when phages equalled or exceeded bacteria (MOI of 1 or higher) population growth was inhibited. Likewise, CRISPR-Cas immunity enabled population growth at low phage doses, but at higher MOIs, the populations either collapsed when infected with ɸM1, or became static when infected with ɸTE (Fig. 4a, Supplementary Fig. 4). We predicted that CRISPR-Cas was providing population-level protection by reducing the phage epidemic. To test this, the effect of CRISPR-Cas on phage titres was determined (Fig. 4b). Both phages replicated extensively on the phage-sensitive WT bacteria, reaching ~10^10^–10^11^ pfu ml^−1^ irrespective of the initial phage dosage (Fig. 4b). ToxIN reduced the population phage burden regardless of the initial phage abundance. CRISPR-Cas immunity limited the phage epidemic when initial viral abundance was low, but when initial phage numbers were higher, CRISPR was unable to suppress the phage burden. In summary, immunity provided by the type I–F CRISPR-Cas system enables population growth under low viral load by reducing virulent phage burden, therefore providing a benefit to the population.

## Discussion

Here we show that the *P. atrosepticum* type I–F CRISPR-Cas system provides immunity against two virulent phages through abortive infection. Abortive infection systems are diverse, being defined phenotypically based on their impact on phages, cells and host populations following infection. Specifically, phage development is impeded, with fewer progeny released and the infected cells die, which provides population-level protection due to the decreased phage burden^19,20^. Indeed, phage-infected cells harbouring CRISPR-Cas immunity did not survive (Fig. 2), but this led to reduced phage propagation (Fig. 1) and the population was protected due to the reduced phage epidemic (Fig. 4).

The proposed dormancy or possible ‘suicidal’ responses upon phage infection in bacteria harbouring some CRISPR-Cas variants, were suggested to occur through several mechanisms^30^. These include activation of toxic domains in some Cas proteins, such as Cas2^31^, collateral ssDNase activity of type V systems^14^, promiscuous RNA-targeting by type III^11–13^ and type VI^10^ systems, and self-targeting due to increased spacer acquisition upon CRISPR-Cas activation^32^. These models cannot explain abortive infection by type I–F system and are ruled out by our CRISPR-Cas plasmid targeting experiments (Fig. 3). Moreover, *P. atrosepticum* Cas2 has no detectable nuclease (i.e. toxic) activity^33^ and although we have observed acquisition of self-targeting spacers, this low frequency is unlikely to significantly impact cell survival^34^. Instead, we propose that CRISPR-Cas immunity still enables a window of time for the virulent phage to express toxic products or takeover host resources and machinery^18^. Although CRISPR-Cas limits phage propagation, the initiation of host-takeover renders cells unable to recover, and abortive infection ensues. Although the mechanism of takeover by ɸM1 and ɸTE is unknown, ɸM1 encodes its own RNAP, suggesting rapid transcriptional reprogramming, and a ɸM1 gene that triggers ToxIN immunity is toxic in *P. atrosepticum*^26^. Therefore, combined with the absence of an Abi phenotype during type I–F plasmid targeting (Fig. 3), our results are entirely consistent with CRISPR-Cas resistance via phage-induced abortive infection.

We hypothesise that the speed and strength of bacterial takeover and the pace and level of CRISPR-Cas immunity will influence the outcome for the infected individual. Indeed, type I–E and I–F CRISPR-Cas systems can also provide phage resistance without apparent Abi phenotypes^16,35^. However, these CRISPR-Cas responses to phages have typically been studied using filamentous phages or virulent mutants of temperate phages (i.e. obligately lytic)^16,35,36^. Temperate phages can transmit both horizontally and vertically and therefore generally avoid immediate early expression of genes involved in host takeover until the lytic-lysogeny decision is made^37^. Temperate mutants locked in an obligately lytic state will initially proceed similarly to their progenitor temperate phages, but inevitably always make the same lytic ‘decision’. Filamentous phages cause chronic infections where bacteria are alive and secrete new phages. For these less ‘aggressive’ phages, CRISPR-Cas may have sufficient time to clear the infection before phage-induced damage becomes irreversible. Whereas in virulent phage infection, early expressed genes can lead to host DNA degradation, inhibition of host RNA polymerase and other effects^38,39^. We propose that in our study, CRISPR-Cas was unable to clear the infection before phage-inflicted cellular damage occurred. This helps explain previous observations that type I–E immunity against T7 or T5 virulent phages slowed or inhibited the growth of *Escherichia coli*^18^.

It is not clear how CRISPR-Cas systems that elicit abortive infection acquire new spacers. However, spacers might be acquired from defective phages^40^ and enable resistant populations to arise in structured environments^21^. Indeed, Abi systems evolve in spatially structured niches where clone mates benefit directly^21,41,42^ and we predict that CRISPR-Cas systems that function through abortive infection will be beneficial under these conditions. Hence, different CRISPR-Cas variants are likely to be beneficial in different ecological settings and will depend on the lifecycle of infecting phages^3^.

Our demonstration that the type I–F system elicits abortive infection broadens the view of how CRISPR-Cas immunity is mediated—sometimes coming at the expense of the individual, but benefitting the population. We propose that the strength and speed of host-takeover by the invader and the relative efficiency of resistance are likely to influence whether CRISPR-Cas provides protection to the infected individual and the population, or just to the population via aborted infection. Therefore, virulent phages are more likely to elicit abortive infection, whereas temperate and filamentous phages, or other mobile genetic elements, will be more likely be cleared. These outcomes may be the extremes of a continuum that is further influenced by temporal factors and invader vs host immune strength, and will need to be factored in to ecological and evolutionary analyses of CRISPR-Cas immunity.

## Methods

### Bacterial strains, plasmids and culture conditions

Bacterial strains and plasmids are listed in Supplementary Table 2. *P. atrosepticum* SCRI1043^43^ was grown at 25 °C and *Escherichia coli* at 37 °C in Lysogeny Broth (LB) at 180 rpm or on LB-agar (LBA) plates containing 1.5% (w v^−1^) agar. Minimal media contained 40 mM K_2_HPO_4_, 14.6 mM KH_2_PO_4_, 0.4 mM MgSO_4_, 7.6 mM (NH_4_)_2_SO_4_ and 0.2% (w v^−1^) glycerol. When required, media were supplemented with ampicillin (Ap, 100 µg ml^−1^), kanamycin (Km; 50 µg ml^−1^), isopropyl-ß-D-thiogalactopyranoside (IPTG, 0.1 mM), glucose (glu, 0.2% (v v^−1^)) and arabinose (ara, 0.2% (v v^−1^)). Bacterial growth was measured in a Jenway 6300 spectrophotometer at 600 nm (OD_600_). All experiments were performed in a minimum of biological triplicates and data shown are the mean + standard deviation.

### Phage storage and titration

The phages, ɸTE^25^ (genome size of ~142 kb) and ɸM1^26,44^ (genome size of ~43 kb), were stored in phage buffer (10 mM Tris-HCl pH 7.4, 10 mM MgSO_4_ and 0.01% w v^−1^ gelatin). Phage stocks were titrated by serially diluting phages in phage buffer, adding to 100 µl of *P. atrosepticum* culture (pre-grown in 5 ml LB overnight) in 4 ml top LBA (0.35% (ɸTE) and 0.5% (ɸM1) agar) and pouring onto LBA plates. Plates were incubated at 25 °C overnight, plaques were counted and the titre determined as plaque forming units (pfu) ml^−1^. Efficiency of plating (EOP) was calculated as: (pfu ml^−1^ (test strain)/pfu ml^−1^ (control strain, *P. atrosepticum*)). For the following assays (excluding the assays with the crRNA and Cas overexpression), strains carried the vector, pBR322, to control for ToxIN (which is on the pBR322 derivative, pTA46).

### Efficiency of centre of infection assays (ECOI)

Overnight cultures were OD-adjusted and 1 ml was used to inoculate a 25 ml culture in a 250 ml flask, for a starting OD_600_ of 0.1. Cells were grown until early stationary phase (OD_600_ of ~0.3) before 10^9^ total phages (~4 × 10^7^ pfu ml^−1^) were added at a multiplicity of infection (MOI) of ~0.1 and cultures were incubated with shaking for 20 min. Aliquots of 1 ml were extracted, washed twice in 1 × phosphate-buffered saline (PBS), diluted and plated in top LBA with *P. atrosepticum* before the infected cells starting lysing. The pfu ml^−1^ was determined for each strain and since each plaque was formed from the phages released from an individual cell, the titre represents the number of infectious centres formed. The ECOI was calculated as (pfu ml^−1^ (test strain)/pfu ml^−1^ (control strain, *P. atrosepticum*)). Spontaneous ɸ-resistant surface mutants, PCF333 and PCF334, were included to control for unadsorbed phages.

### One-step growth curves

Overnight cultures were OD-adjusted and 1 ml was used to inoculate a 25 ml culture in a 250 ml flask, for a starting OD_600_ of 0.1. Cells were grown until early exponential phase (OD_600_ of 0.25–0.35) and 10^9^ total phages (~4 × 10^7^ pfu ml^−1^) were added, for an MOI of ~0.1. Duplicate samples were taken at various timepoints, until 70 min post infection. One sample was plated immediately (non-treated sample, free phages and phage-infected cells), while the second was added to phage buffer containing chloroform (treated sample, free phages and phage accumulated inside infected cells), which lysed the cells, allowing the assessment of the total number of mature phages at each time point. Samples were diluted in phage buffer and plated in top LBA with *P. atrosepticum*. Phage adsorption over time was determined from the treated samples using the equation ((pfu ml^−1^ (t = 0) − pfu ml^−1^ (t = 0–70)/pfu ml^−1^ (t = 0)). The average phage burst size was also calculated from the treated samples, as number of phages released ((pfu ml^−1^ (t = 70) − pfu ml^−1^ (t = 30))/the number of cells infected ((pfu ml^−1^ (t = 0) − pfu ml^−1^ (t = 30)). The latent period was determined from the treated samples as was defined as the time before the phage burst starts.

### Cell survival assays

Cells were grown to OD_600_ ~ 0.3 and for each culture, 1 ml was transferred into two universals. One culture was infected with phages at a MOI of ~2, while the other was mock infected, with phage buffer. Cultures were shaken at 180 rpm for 20 min for phages to adsorb and then cells were pelleted and resuspended in PBS to remove unadsorbed phages. Finally, cells were diluted and 100 µl samples were plated prior to the phage burst (40 min). Cell survival was calculated as (colony forming units (cfu) ml^−1^ (phage treated sample)/cfu ml^−1^ (mock treated sample).

To assess cell survival at a range of MOIs, 100 µl of each exponential phase culture was aliquoted into eight wells of a 96-well flat-bottomed plate for the addition of 10 µl phages at seven MOIs as well as a mock infection control (phage buffer). Cultures were shaken for 20 min for phages to adsorb, and to reduce the burden of secondary infection, a viricidal solution called TEAF (per ml: 680 µl of 4.3 mM FeS0_4,_ 320 µl 7.5% (w v^−1^) green tea solution (filter-sterilised)^45^) was then added, at a ratio of 75% (v v^−1^) to each culture. The cultures were then diluted, more TEAF was added to each dilution and cells were plated as 5 µl spots. Survival for the ɸTE-infected cells was higher than predicted from the MOIs used, suggesting that despite high adsorption rates (Supplementary Table 1), the phage was not able to infect as well in these 96-well assays with high phage doses.

### LIVE/DEAD staining for membrane activity

Cell membrane integrity was assessed using the LIVE/DEAD^™^ *Bac*Light^™^ bacterial viability kit, consisting of two nucleic acid stains, syto-9 and propidium iodide (Life technologies^™^). Cultures were prepared for LIVE/DEAD^™^ staining as described above for the cell survival assays performed at a range of MOIs. Cells were infected for one hour, to allow for one complete round of infection, before being stained, according to the manufacturers’ instructions. Culture fluorescence was measured using a Thermo Scientific^™^ Varioskan^™^ plate reader, with excitation/emission wavelengths of 485/530 nm for styo-9 and 485/630 nm for propidium iodide. Cultures of exponentially growing cells and cells killed with 70% isopropanol were combined at different ratios to generate a standard curve, from which the percentage of cells with intact membranes at each phage MOI could be determined.

### Resazurin assays for cell activity

For assays assessing cell activity after one round of phage infection, cultures were prepared as described above for the cell survival assays performed at a range of MOIs. Cells were infected for one hour before resazurin solution was added at a final concentration of 0.005% (w v^−1^). Cellular oxidoreductases reduce the blue indicator to resorufin, which is pink. Resorufin fluorescence was measured 30 min after it was added using a Thermo Scientific^™^ Varioskan^™^ plate reader with excitation/emission wavelengths of 510/535 nm. Cells for the standard curve were prepared as described for the LIVE/DEAD^™^ staining, from which the percentage of metabolically active cells at each MOI was determined. Cell activity was assessed, following the 16 h growth assays, in the same way.

### Isolation of spontaneous phage-resistant surface mutant strains

ɸTE and ɸM1 were plated on *P. atrosepticum* and cells from colonies that formed in the centre of plaques were streaked to single colonies. Since ɸTE is flagella-trophic^46^, clones isolated from plates with ɸTE were patched onto tryptic swimming agar (10 g Bacto tryptone, 5 g NaCl, 3 g agar, per litre) to assess flagella-mediated swimming. A clone that did not swim (PCF333, Supplementary Table 2) was resistant to ɸTE, but sensitive to ɸM1, which does not use the flagella for infection, suggesting that it was a surface mutant. A clone isolated from a ɸM1 plaque (PCF334, Supplementary Table 2) was ɸM1-resistant, but sensitive to ɸTE.

### Construction of the plasmids expressing crRNAs

Spacers present in strains targeting ɸTE (PCF190) and ɸM1 (PCF254) were cloned into pPF975. Overlapping primers containing the spacer sequences were annealed and ligated into the BsaI site in the mini-CRISPR array (repeat-repeat loci) as previously described^47^ to form the plasmids, pPF1421 and pPF1423 (Supplementary Table 2). Oligonucleotide sequences are listed in Supplementary Table 3. All plasmids used in this study were confirmed by sequencing. In assays using these plasmids, their expression was induced with IPTG. 

### Construction of the *cas* overexpression strains

The chromosomal *cas* overexpression strain (PCF610) was made by conjugating the suicide vector, pPF1814, into *P. atrosepticum*. The vector, pPF1814 was constructed as follows: pSEVA511 was digested with NotI and ligated with the T5/*lac* promoter and multiple cloning site (MCS) from pQE-80L-stuffer, which had been amplified with PF3494 and PF3495 and digested with NotI. The *lacI* gene was amplified from pQE-80L-stuffer (PF2511, PF2512) and ligated into the MCS at XmaI and SalI sites. Finally, the first 500 bp of *cas1* was amplified using PF357 and PF669 and ligated into EcoRI and XmaI sites in the MCS. In assays using these strains, their expression was induced with IPTG.

### Plasmid targeting assay

The effect of plasmid targeting on cell survival was assessed using a two-plasmid setup. The first plasmid was either a control vector (pControl, pPF445, Ap^R^) with an inducible mini-CRISPR array with a single repeat or pCRISPR (pPF452, Ap^R^) carrying a spacer targeting *expI*. The second plasmid was pTargeted (pPF459, Km^R^), which carried the targeted *expI* gene. pTargeted was made by PCR-amplifying *expI* from *P. atrosepticum* with PF314 and PF317, digesting the product with BamHI and PstI and ligating the product into the same sites in pPF260 (Km^R^-pQE-80L derivative). pControl and pCRISPR were made previously^48^. *P. atrosepticum* Δ*expI* (PCF81) was co-transformed with pTargeted and pCRISPR, or pControl, under CRISPR repressing conditions (0.2% glu) with both antibiotics (Km and Ap). These strains were for 6 h in LB, 0.2% glu, Ap + Km with shaking. Cells were pelleted by centrifugation, washed and the culture was split into two samples, repressed (0.2% glu and Ap) and induced CRISPR conditions (0.2% ara and Ap). Following growth for a further 18 h, cells were plated onto Ap (for total cell counts) and Km (for targeted vector-containing cell counts). Efficiency of plasmid maintenance was calculated from the Km counts as (cfu ml^−1^ (pCRISPR)/cfu ml^−1^ (pControl)). Cell survival was calculated for each strain as (cfu ml^−1^ (induced)/cfu ml^−1^ (repressed). The cell counts for the induced CRISPR conditions were higher because the growth rate of *P. atrosepticum* was increased with supplemented arabinose.

### Bacterial population growth assays

*P. atrosepticum* cultures were grown to an OD_600_ of 0.3 and 100 μl was transferred to each well (of a 96-well plate). Phages were added in 10 μl at multiplicities of infection (MOIs) ranging from 0.0001 to 10 and cultures were grown in a Thermo Scientific^™^ Varioskan^™^ plate reader with shaking at 480 rpm. Cell density was monitored for 16 h, measuring OD_600_ every 12 min. Following growth, final phage titres were determined by chloroform treating the bacterial cultures and titrating the phages. The data were processed using GraphPad Prism to generate restricted cubic spline curves (324 points were calculated).

### Reporting summary

Further information on research design is available in the Nature Research Reporting Summary linked to this article.

## Supplementary information

Supplementary Information

Reporting Summary

## Data Availability

The source data underlying Figs. 1–4 and Supplementary Figs. 1–4 are provided as a Source Data file. The data that support the findings of this study are available in this article and its Supplementary Information files, or from the corresponding author upon request.

## Source Data

Source Data
