# Supplementary material for: Type I-F CRISPR-Cas resistance against virulent phages results in abortive infection and provides population-level immunity
Source: Nat Commun. 2019 Dec 4;10:5526. doi: 10.1038/s41467-019-13445-2 (PMC6892833; doi:10.1038/s41467-019-13445-2)
Supplement: Supplementary file 1 — Supplementary Information [file 41467_2019_13445_MOESM1_ESM.pdf]

## Supplementary Information

# Type I-F CRISPR-Cas resistance against virulent phages results in abortive infection and provides population-level immunity

Bridget N.J. Watson[1#], Reuben B. Vercoe[1], George P.C. Salmond[2], Edze R. Westra[3], Raymond H.J Staals[1,4] and Peter C. Fineran[1,5*]

[1]Department of Microbiology and Immunology, University of Otago, PO Box 56, Dunedin 9054, New Zealand.

[2]Department of Biochemistry, University of Cambridge, Cambridge CB2 1QW, United Kingdom.

[3]ESI, Biosciences, University of Exeter, Cornwall Campus, Penryn TR10 9FE, UK.

[4]Laboratory of Microbiology, Wageningen University and Research, 6708 WE Wageningen, The Netherlands

[5]Bio-Protection Research Centre, University of Otago, Dunedin, New Zealand.

[#]Present address: ESI, Biosciences, University of Exeter, Cornwall Campus, Penryn TR10 9FE, UK.

*For correspondence: peter.fineran@otago.ac.nz

## Supplementary Figures

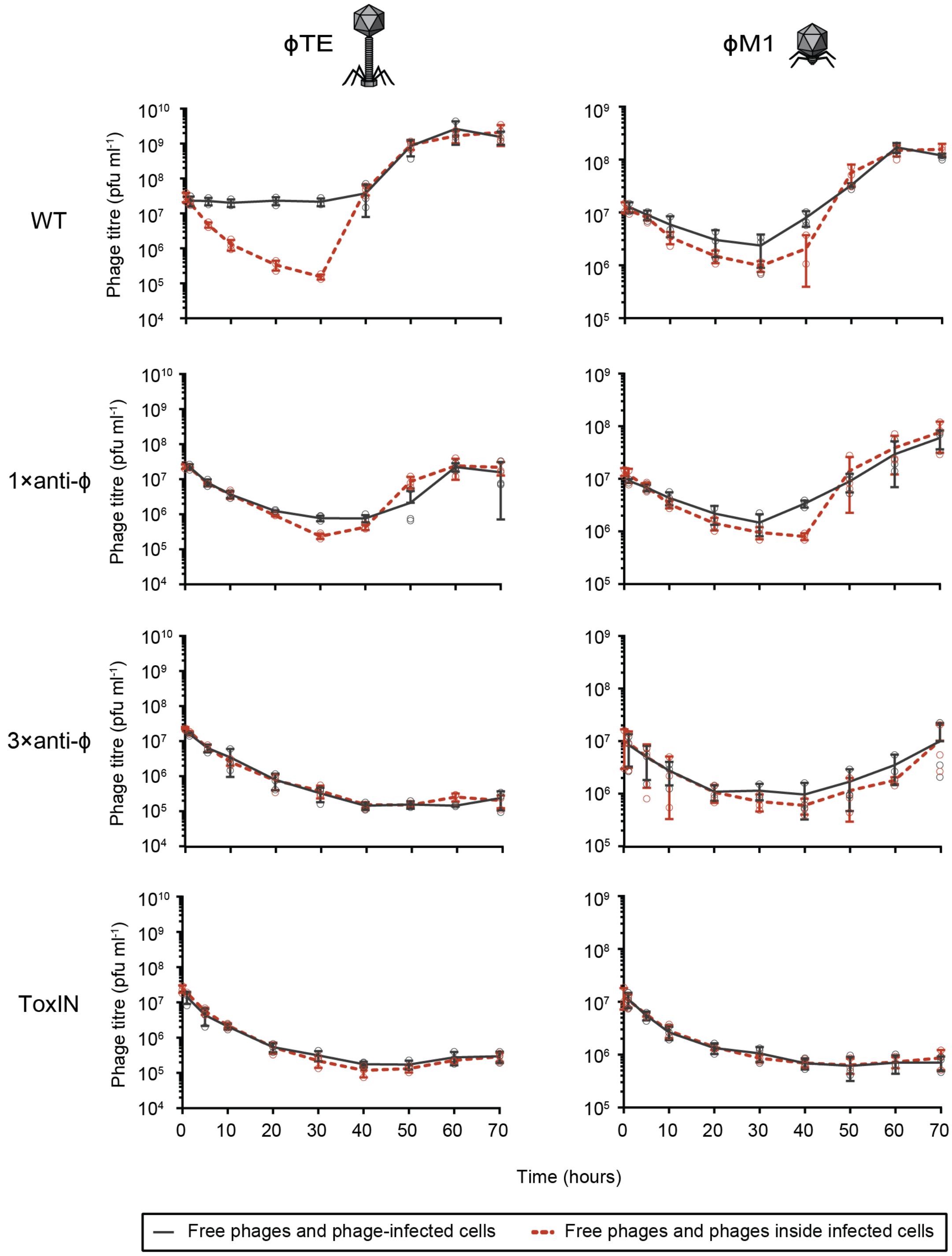


**Supplementary Figure 1. One-step growth curves provide insight into adsorption and phage burst size.** Assays were performed on strains infected at an MOI of ~0.1. Samples were non-treated (free phages and phage-infected cells, black line) or treated with chloroform (free phages and phages accumulated inside infected cells, red dashed line). In the ϕTE infected WT cells, the red line decreases since phages have adsorbed to the cell and have injected their DNA. As these samples are treated with chloroform to lyse the cells these 'infecting' phages are not seen as plaques. The black line does not go down, since the 'infecting' phages can continue replicating once the intact cells are plated. Strains with immunity mediated by CRISPR-Cas or ToxIN do not show this trend as there is reduced phage replication. Phage burst size and adsorption data was calculated for Fig. 1 and Table S1. Source data are provided as a Source Data file.

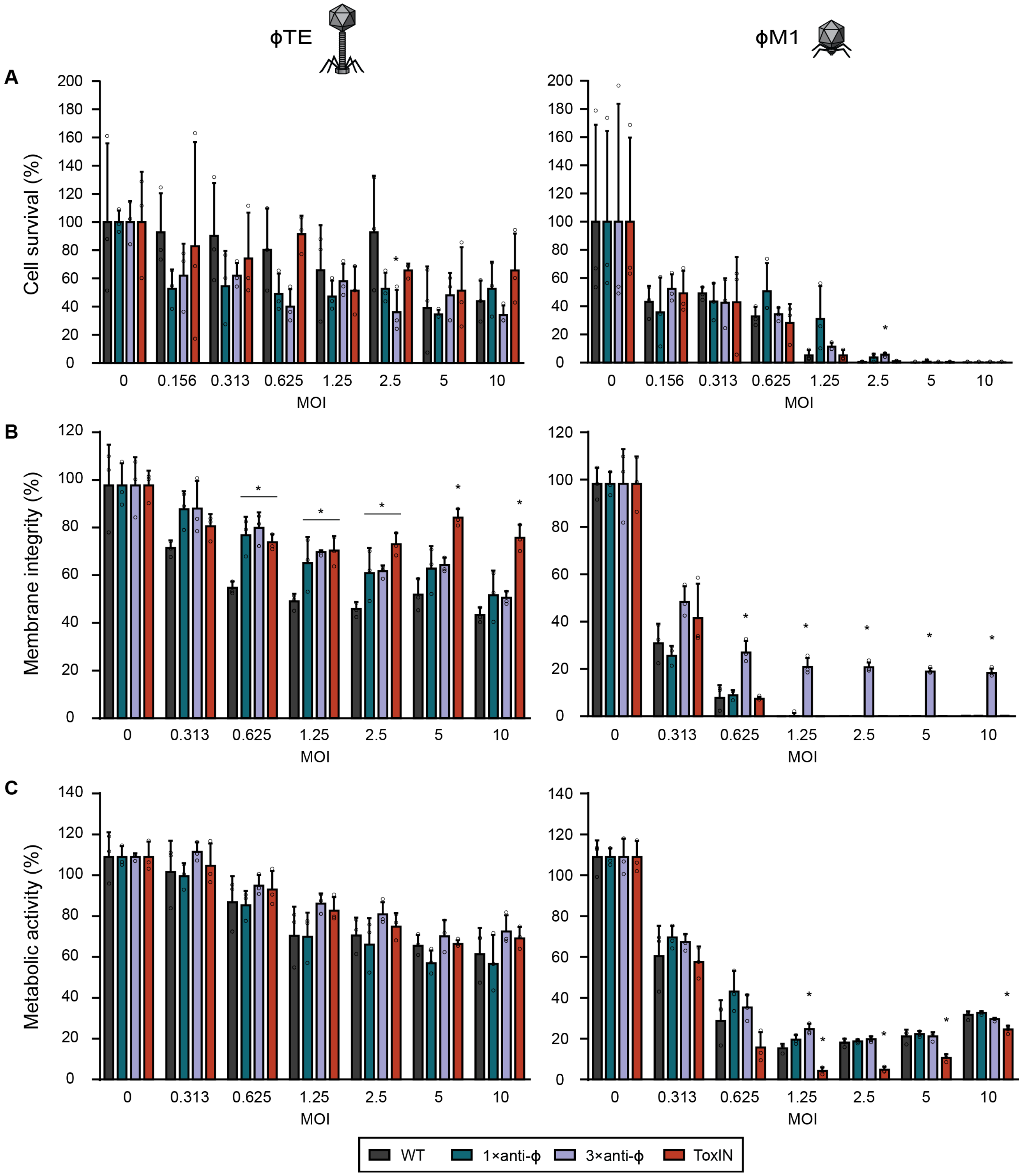


**Supplementary Figure 2. CRISPR-Cas immunity does not promote cell survivial at a range of MOIs. A** Cell survival, **B** membrane integrity and **C** metabolic activity was assessed at a range of MOIs for WT, 1× and 3×anti-ϕ strains and ToxIN, using both ϕTE and ϕM1. Statistical significance was calculated using one-way ANOVA using Dunnett's multiple comparison test, comparing strains with targeting spacers to the control with no-targeting spacers. No significance was detected, unless indicated (* $p \leq 0.05$). Source data are provided as a Source Data file.

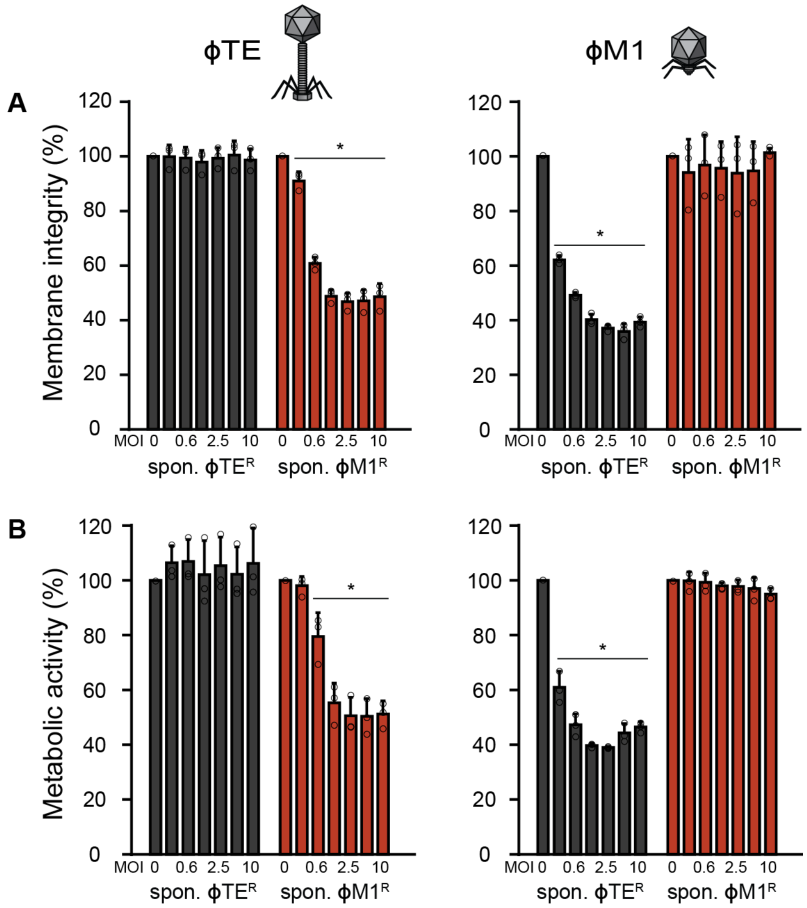


**Supplementary Figure 3. Spontaneous phage-resistant mutants are active in the presence of phages.** Spontaneous ϕTE$^{R}$ and ϕM1$^{R}$ mutants were infected with ϕTE and ϕM1 at different MOIs (0, 0.3, 0.6, 1.25, 2.5, 5 and 10) and **A** membrane integrity **B** cell activity levels was assessed following one round of infection. Statistical significance was calculated using one-way ANOVA using Dunnett's multiple comparison test, comparing the phage-infected samples to the uninfected sample for each strain. No significance was detected, unless indicated (* $p \leq 0.05$). Source data are provided as a Source Data file.

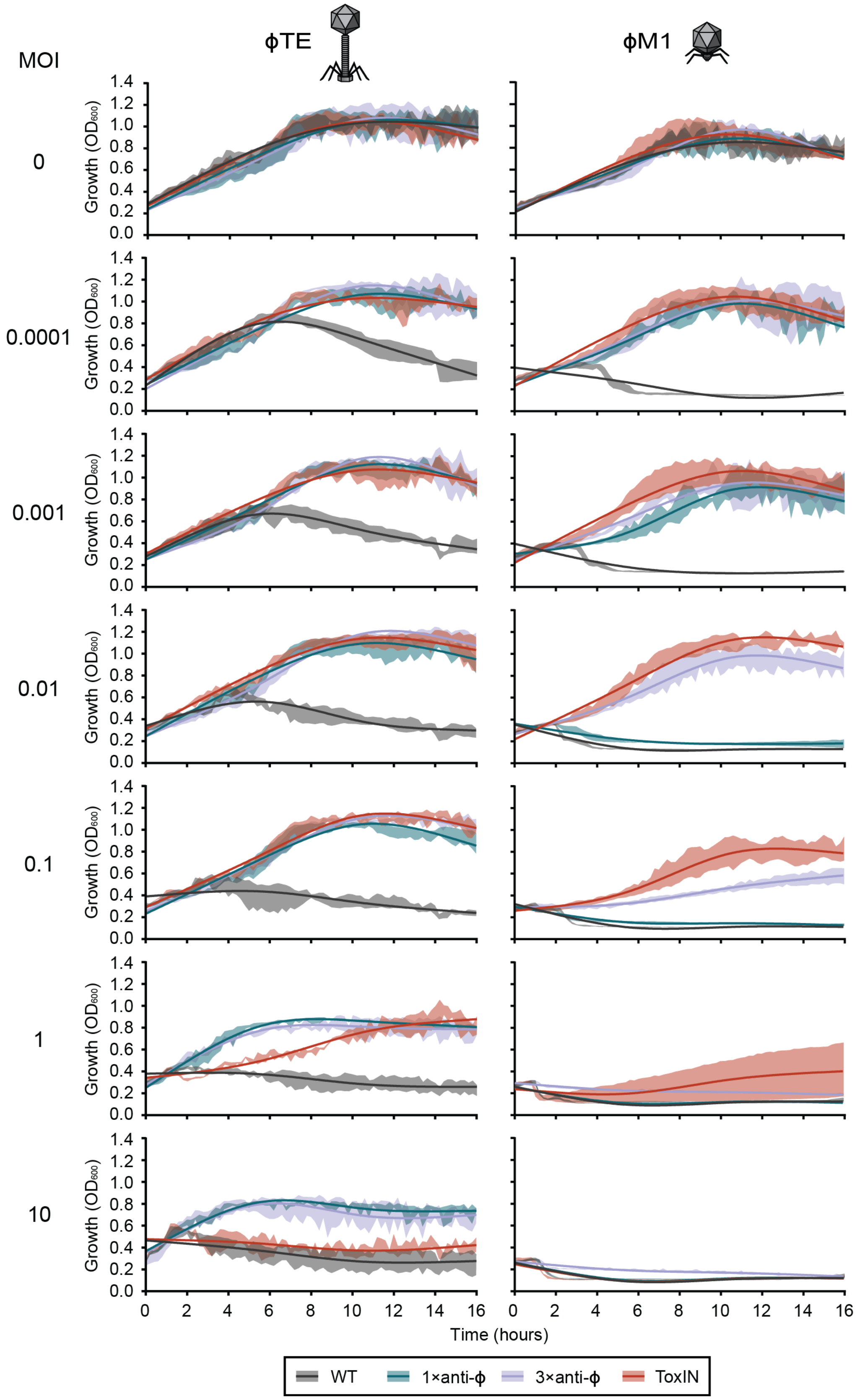


**Supplementary Figure 4. Anti-ϕ strains grow in the presence of phages up to a MOI of 1.** Strains were grown in the presence of phages at different MOIs and $OD_{600}$ measurements were taken every 12 min for 16 hours. Solid lines: restricted cubic spline curve of the $OD_{600}$ values, shaded colour: one SD of the mean $OD_{600}$. These are the full data from what is presented in Fig. 4. Source data are provided as a Source Data file.

## Supplementary Tables

**Supplementary Table 1.** Characteristics of phages ϕTE and ϕM1.

| Phage/ host | EOP | ECOI (%) | Latent period (min) | Adsorption (%) | Burst size (phages) |
|---|---|---|---|---|---|
| **ϕTE** | | | | | |
| WT | $1.0\times10^{0} \pm 6.0\times10^{-2}$ | 100 ±18.7 | 30 ±0 | 99 ±0.00 | 75 ±44 |
| 1×anti-ϕTE | $2.0\times10^{-3} \pm 1.2\times10^{-3}$ | 4.1 ±1.6 | 33 ±6 | 99 ±0.00 | 1 ±0 |
| 3×anti-ϕTE | $1.1\times10^{-5} \pm 5.6\times10^{-6}$ | 0.9 ±0.3 | n/d | 98 ±0.01 | <1 |
| ToxIN | $1.9\times10^{-6} \pm 4.1\times10^{-7}$ | 1.1 ±0.4 | n/d | 99 ±0.00 | <1 |
| | | | | | |
| **ϕM1** | | | | | |
| WT | $1.0\times10^{0} \pm 4.2\times10^{-1}$ | 100 ±50.7 | 37 ±6 | 92 ±0.04 | 13 ±3 |
| 1×anti-ϕM1 | $1.5\times10^{-1} \pm 6.5\times10^{-2}$ | 22.5 ±17.4 | 37 ±6 | 93 ±0.03 | 6 ±3 |
| 3×anti-ϕM1 | $4.7\times10^{-3} \pm 2.1\times10^{-4}$ | 6.3 ±3.5 | 40 ±0 | 90 ±0.08 | 1 ±0 |
| ToxIN | $2.3\times10^{-5} \pm 7.4\times10^{-6}$ | 1.5 ±0.6 | n/d | 93 ±0.02 | <1 |

Data shown is the mean ±SD. n/a not applicable. n/d no data the pfu values continue to decrease and there was no detectable phage burst.

**Supplementary Table 2.** Bacterial strains and plasmids used in this study.

| Strain/Plasmid | Relevant Genotype/Phenotype | Reference |
|---|---|---|
| **Strains** | | |
| ***Escherichia coli*** | | |
| DH5α | F-, φ80Δ*dlacZ*M15, Δ(*lacZYA–argF*)U169, *endA1*, *recA1*, *hsd*R17 ($r_K^- m_K^+$), *deoR*, *thi-1*, *supE*44, λ-, *gyr*A96, *rel*A1 | Gibco/BRL |
| ST18 | *recA, pro, hsdR, recA*::RP4-2-Tc::Mu, λ*pir*, $Tmp^R$, $Sp^R$, $Sm^R$, Δ*hemA* | [1] |
| ***Pectobacterium atrosepticum*** | | |
| SCRI1043 | Wild type (WT) | [2] |
| PCF81 | SCRI1043 Δ*expI*::*cat*, $Cm^R$ | [3] |
| PCF188 | SCRI1043 with 3x anti-ϕTE spacers (in CRISPR1+2) | [4] |
| PCF190 | SCRI1043 with 1x anti-ϕTE spacer (in CRISPR1) | [5] |
| PCF254 | SCRI1043 with 1x anti-ϕM1 spacer (in CRISPR1) | [5] |
| PCF256 | SCRI1043 with 3x anti-ϕM1 spacers (in CRISPR1+2) | [5] |
| PCF333 | SCRI1043 with spontaneous $ϕTE^R$ | This study |
| PCF334 | SCRI1043 with spontaneous $ϕM1^R$ | This study |
| PCF610 | SCRI1043 with integrated pPF1814 for *cas* operon overexpression | This study |
| **Plasmids** | | |
| pBR322 | Cloning vector, ColE1 ori, $Tc^R$, $Ap^R$ | [6] |
| pPF260 | pQE-80L derivative with RP4 oriT, $Km^R$ | [7] |
| pPF445 (“pControl”) | mini-CRISPR with 1 repeat, pBAD30-derivative (aka pC1-16), p15a ori, $Ap^R$ | [3] |
| pPF452 (“pCRISPR”) | mini-CRISPR with single spacer targeting *expI*, pPF445 - derivative (aka pE1-16)), $Ap^R$ | [3] |
| pPF459 (“pTargeted”) | pPF260-derivative with *P. atrosepticum expI* gene, $Km^R$ | This study |
| pPF975 | pPF260-derivative, IPTG-inducible CRISPR locus for expressing crRNAs, $Km^R$ | [8] |
| pPF1421 | pPF975-derivative with the spacer from PCF254 | This study |
| pPF1423 | pPF975-derivative with the spacer from PCF190 | This study |
| pPF1814 | pSEVA511-derivative with T5/*lac* promoter, MCS and *lacI* from pQE-80L-stuffer, and 500 bp of *cas1* | This study |
| pQE-80L-stuffer | pQE-80L (Qiagen) with the 6His removed by digestion with EcoRI and BamHI and these sites restored, $Ap^R$ | Josh Ramsay; unpublished |
| pSEVA511 | R6K ori, $Tc^R$ | [9] |
| pTA46 | pBR322-derivative containing *toxIN*, $Ap^R$ | [10] |

**Supplementary Table 3.** Oligonucleotide sequences used in this study

| Name | Sequence (5'-3') | Description |
|---|---|---|
| PF210 | GTCATTACTGGATCTATCAACAGG | R 100 bp downstream of CRISPR locus in pPF975 |
| PF314 | TTTGGTACCGGATCCGTGGCAATGATTACTCCATC | F for amplifying *expI* from *P. atrospeticum* (BamHI) |
| PF317 | TTTTCTAGACTGATGAATGGGTGAATCTC | R for amplifying *expI* from *P. atrospeticum* (XbaI) |
| PF357 | GACGAATTCTTACGGAAGAAAATACATTATGG | F for amplifying *cas1* N-terminal (EcoRI) |
| PF669 | TTTCCCGGGAAAGGTAAAGCGCGATTCAC | R for amplifying 500 bp into *cas1* (XmaI) |
| PF2511 | TCTCCCGGGAGGCATCAAATAAAACGA | F for amplifying *lacI* from pQE-80L (XmaI) |
| PF2512 | TCTGTCGACACACCATCGAATGGTGCA | R for amplifying *lacI* from pQE-80L (SalI) |
| PF2565 | GAAAACTAGCGTCTGTAGTGGGTCGTTGTGCAAGTAG | F for cloning PCF254 spacer into pPF975 |
| PF2566 | TGAACTACTTGCACAACGACCCACTACAGACGCTAGT | R for cloning PCF254 spacer into pPF975 |
| PF2569 | GAAATGACACAGCCAACGCCCTGAAAATCGGCACAGG | F for cloning PCF190 spacer into pPF975 |
| PF2570 | TGAACCTGTGCCGATTTTCAGGGCGTTGGCTGTGTCA | R for cloning PCF190 spacer into pPF975 |
| PF3494 | TTTGCGGCCGCTCGTCTTCACCTCGAGAAATC | F for amplifying pQE-80L MCS (NotI) |
| PF3495 | TTTGCGGCCGCGTCATTACTGGATCTATCAACAGG | R for amplifying pQE-80L MCS (NotI) |

## References


1 Thoma, S. & Schobert, M. An improved Escherichia coli donor strain for diparental mating. *FEMS microbiology letters* **294**, 127-132, doi:10.1111/j.1574-6968.2009.01556.x (2009).

2 Bell, K. S. *et al.* Genome sequence of the enterobacterial phytopathogen *Erwinia carotovora* subsp. *atroseptica* and characterization of virulence factors. *Proc Natl Acad Sci U S A* **101**, 11105-11110, doi:10.1073/pnas.0402424101 (2004).

3 Vercoe, R. B. *et al.* Cytotoxic chromosomal targeting by CRISPR/Cas systems can reshape bacterial genomes and expel or remodel pathogenicity islands. *PLoS Genet* **9**, e1003454, doi:10.1371/journal.pgen.1003454 (2013).

4 Pawluk, A. *et al.* Inactivation of CRISPR-Cas systems by anti-CRISPR proteins in diverse bacterial species. *Nature Microbiology* **1**, 16085, doi:10.1038/nmicrobiol.2016.85 (2016).

5 Watson, B. N. J., Staals, R. H. J. & Fineran, P. C. CRISPR-Cas-mediated phage resistance enhances horizontal gene transfer by transduction. *mBio* **9**, e02406-02417, doi:10.1128/mBio.02406-17 (2018).

6 Bolivar, F. *et al.* Construction and characterization of new cloning vehicle. II. A multipurpose cloning system. *Gene* **2**, 95-113, doi:10.1016/0378-1119(77)90000-2 (1977).

7 Richter, C. *et al.* Priming in the Type I-F CRISPR-Cas system triggers strand-independent spacer acquisition, bi-directionally from the primed protospacer. *Nucleic Acids Res* **42**, 8516-8526, doi:10.1093/nar/gku527 (2014).

8 Jackson, S. A., Birkholz, N., Malone, L. M. & Fineran, P. C. Imprecise Spacer Acquisition Generates CRISPR-Cas Immune Diversity through Primed Adaptation. *Cell Host Microbe* **25**, 250-260 e254, doi:10.1016/j.chom.2018.12.014 (2019).

9 Silva-Rocha, R. *et al.* The Standard European Vector Architecture (SEVA): a coherent platform for the analysis and deployment of complex prokaryotic phenotypes. *Nucleic acids research* **41**, D666-675, doi:10.1093/nar/gks1119 (2013).

10 Fineran, P. C. *et al.* The phage abortive infection system, ToxIN, functions as a protein-RNA toxin-antitoxin pair. *Proc Natl Acad Sci U S A* **106**, 894-899, doi:10.1073/pnas.0808832106 (2009).
